# INTEGRATING VIRTUAL REALITY DEMENTIA SENSITIVITY TRAINING INTO THE UNDERGRADUATE CURRICULUM

**DOI:** 10.1093/geroni/igae098.1938

**Published:** 2024-12-31

**Authors:** Tamar Shovali

**Affiliations:** Eckerd College, St Petersburg, Florida, United States

## Abstract

There is a need for Age-Friendly endeavors to include dementia-inclusive educational opportunities in higher education. Given the rising aging population, undergraduate students increasingly will (a) have personal experiences with loved ones diagnosed with dementia or Alzheimer’s disease, (b) pursue careers in the field of aging and directly work with individuals with dementia or Alzheimer’s disease, or (c) indirectly work with individuals with dementia or Alzheimer’s disease and their caregivers through roles in various professions such as finance or law. Undergraduate students in a gerontology course at an Age-Friendly University engaged in a dementia inclusive virtual reality training intended to sensitize individuals to the needs of people living with dementia. Virtual reality experiences related to dementia can help build empathy towards daily challenges faced by people living with dementia. Prior to and after engagement in the session students were asked questions about their understanding and empathy for people with dementia and their caregivers. Small group discussions followed the session to prompt reflection, encourage critical thinking, and facilitate discussion on the impact and lessons learned from the training. This experiential learning was preceded by an interactive lecture about the basics of Alzheimer’s disease and dementia delivered by a Community Educator from the Alzheimer’s Association. Age-Friendly University principles provide a guideline for the many ways higher education can shape age inclusive teaching and learning environments that promote greater empathy and understanding for people living with dementia or Alzheimer’s disease and their caregivers.

